# Multiplex Antibody Analysis of IgM, IgA and IgG to SARS-CoV-2 in Saliva and Serum From Infected Children and Their Close Contacts

**DOI:** 10.3389/fimmu.2022.751705

**Published:** 2022-01-27

**Authors:** Carlota Dobaño, Selena Alonso, Marta Vidal, Alfons Jiménez, Rocío Rubio, Rebeca Santano, Diana Barrios, Gemma Pons Tomas, María Melé Casas, María Hernández García, Mònica Girona-Alarcón, Laura Puyol, Barbara Baro, Pere Millat-Martínez, Sara Ajanovic, Núria Balanza, Sara Arias, Natalia Rodrigo Melero, Carlo Carolis, Aleix García-Miquel, Elisenda Bonet-Carné, Joana Claverol, Marta Cubells, Claudia Fortuny, Victoria Fumadó, Anna Codina, Quique Bassat, Carmen Muñoz-Almagro, Mariona Fernández de Sevilla, Eduard Gratacós, Luis Izquierdo, Juan José García-García, Ruth Aguilar, Iolanda Jordan, Gemma Moncunill

**Affiliations:** ^1^ ISGlobal, Hospital Clínic - Universitat de Barcelona, Barcelona, Spain; ^2^ Consorcio de Investigación Biomédica en Red (CIBER) de Enfermedades Infecciosas, Madrid, Spain; ^3^ Consorcio de Investigación Biomédica en Red de Epidemiología y Salud Pública (CIBERESP), Madrid, Spain; ^4^ Pediatrics Department, Hospital Sant Joan de Déu, Universitat de Barcelona, Esplugues, Spain; ^5^ Institut de Recerca Sant Joan de Déu, Esplugues, Spain; ^6^ Paediatric Intensive Care Unit, Hospital Sant Joan de Déu, Universitat de Barcelona, Barcelona, Spain; ^7^ Biomolecular Screening and Protein Technologies Unit, Centre for Genomic Regulation (CRG), The Barcelona Institute of Science and Technology, Barcelona, Spain; ^8^ Fetal Medicine Research Center (Hospital Clínic and Hospital Sant Joan de Déu), Universitat de Barcelona, Barcelona, Spain; ^9^ Institut d’Investigacions Biomèdiques August Pi i Sunyer (IDIBAPS), Barcelona, Spain; ^10^ Universitat Politècnica de Catalunya, BarcelonaTech, Barcelona, Spain; ^11^ Fundació Sant Joan de Déu, Barcelona, Spain; ^12^ Infectious Diseases Department, Hospital Sant Joan de Déu, Barcelona, Spain; ^13^ Biobank Hospital Sant Joan de Déu, Barcelona, Spain; ^14^ Centro de Investigação em Saúde de Manhiça (CISM), Maputo, Mozambique; ^15^ Catalan Institution for Research and Advanced Studies (ICREA), Barcelona, Spain; ^16^ Department of Medicine, Universitat Internacional de Catalunya, Barcelona, Spain; ^17^ Molecular Microbiology Department, Hospital Sant Joan de Déu, Esplugues, Spain; ^18^ Center for Biomedical Research on Rare Diseases (CIBER-ER), Madrid, Spain

**Keywords:** SARS-CoV-2, COVID-19, saliva, antibody - antigen complex, children, plasma, serum, surveillance

## Abstract

COVID-19 affects children to a lesser extent than adults but they can still get infected and transmit SARS-CoV-2 to their contacts. Field deployable non-invasive sensitive diagnostic techniques are needed to evaluate the infectivity dynamics of SARS-CoV-2 in pediatric populations and guide public health interventions, particularly if this population is not fully vaccinated. We evaluated the utility of high-throughput Luminex assays to quantify saliva IgM, IgA and IgG antibodies against five SARS-CoV-2 spike (S) and nucleocapsid (N) antigens in a contacts and infectivity longitudinal study in 122 individuals (52 children and 70 adults). We compared saliva versus serum/plasma samples in infected children and adults diagnosed by weekly RT-PCR over 35 days (n=62), and those who consistently tested negative over the same follow up period (n=60), in the Summer of 2020 in Barcelona, Spain. Saliva antibody levels in SARS-CoV-2 RT-PCR positive individuals were significantly higher than in negative individuals and correlated with those measured in sera/plasmas. Asymptomatic infected individuals had higher levels of anti-S IgG than symptomatic individuals, suggesting a protective anti-disease role for antibodies. Higher anti-S IgG and IgM levels in serum/plasma and saliva, respectively, in infected children compared to infected adults could also be related to stronger clinical immunity in them. Among infected children, males had higher levels of saliva IgG to N and RBD than females. Despite overall correlation, individual clustering analysis suggested that responses that may not be detected in blood could be patent in saliva, and vice versa.

In conclusion, measurement of SARS-CoV-2-specific saliva antibodies should be considered as a complementary non-invasive assay to serum/plasma to determine COVID-19 prevalence and transmission in pediatric populations before and after vaccination campaigns.

## Introduction

Since the start of the COVID-19 pandemic, it has become apparent that pediatric populations are less affected than adult or older populations (1, 2), although studies have also found children and adults to be infected to a similar low degree (3). Clinical presentation of SARS-CoV-2 infection is milder in children, with more proportion of asymptomatic cases (1, 4–6). One hypothesis for this lower severity of COVID-19 is the protective effect that antibodies from human coronaviruses of the common cold (HCoV), which are more prevalent in children, could exert on SARS-CoV-2 control, considering cross-reactivity between them (7, 8). Children could also be harboring lower viral loads, in part due to the lower expression of ACE2, receptor of the coronavirus (9). This implies that highly sensitive techniques might be required for its accurate diagnosis.

In this context, a question of high interest has been whether children who become infected might be less efficient transmitters to their immediate contacts (10), as this has important implications for the management of outbreaks in schools, extracurricular activities, and holiday camps, particularly when children are not prioritized for vaccination campaigns. Having field deployable diagnostic tools to monitor infectivity dynamics in school-like environments is therefore very relevant from the public health perspective.

The presence of the virus in nasal or nasopharyngeal samples can be detected with real time polymerase chain reaction (RT-PCR) and antigen-based sensitive methods, while prior exposure has to be assessed by detecting antibodies to SARS-CoV-2 antigens. The latter is usually done by measuring specific immunoglobulins in serum or plasma samples, which requires obtaining blood samples by venous or capillary punctures, less amenable to large field studies. Several commercial rapid diagnostic tests (RDT) exist that are useful as point of care kits, but they may be limited by their sensitivity and specificity in the case of asymptomatic infections, which usually induce lower levels of antibodies (11, 12). A number of laboratory-based serological assays (ELISA, CLIA) are also widely used and with good performances (13). The pooled sensitivity of ELISAs measuring IgG or IgM has been reported as 84.3% (95% confidence interval 75.6% to 90.9%), and of CLIAs as 97.8% (46.2% to 100%). Pooled specificities ranged from 96.6% to 99.7%. Similarly, multiplex assays combining multiple antigenic specificities simultaneously and amenable for high-throughput testing, offer even better potential to have the highest sensitivity to detect low-level responses in younger populations.

Being a respiratory pathogen, the mucosal immunity has a key importance, and thus the role of IgA in controlling the virus is becoming increasingly important (14). Therefore, saliva is an attractive sample matrix for developing field-deployable non-invasive serological assays that are readily applicable in pediatric surveys (15–17). Indeed, antibodies in saliva have been detected in COVID-19 patients and correlate with plasma antibodies (18). Consequently, the availability of highly sensitive and specific antibody assays for immunological profiling is valuable both for immune-epidemiological surveys and to better understand protective immunity to SARS-CoV-2 (19). In addition, saliva serology would allow knowing the serological diagnosis after the vaccination for determining the levels of immunity at individual level and in the population, and could be really helpful in population screening for determining SARS-CoV-2 seroprevalence in children populations.

In this study we adapted and evaluated three Luminex-based antibody assays to quantify the levels of IgM, IgA and IgG against several SARS-CoV-2 antigens in saliva samples, and compared them to the levels of antibodies obtained using serum/plasma samples from the same individuals who had a positive or negative RT-PCR diagnosis over a five week follow up period. We tested the applicability of the Luminex saliva assays in children and adult volunteers, having different demographic characteristics and clinical presentations, participating in a contacts and infectivity study after the first peak of the COVID-19 pandemic in the Summer of 2020 in Barcelona.

## Materials and Methods

### Study Design, Human Subjects and Samples

We compared the levels of SARS-CoV-2 antibodies in subjects with a positive diagnosis by nasopharyngeal RT-PCR and/or ELISA SARS-CoV-2 IgG, IgM test (Euroimmune Architect – Abbott) independently of the presence of COVID-19 compatible symptoms and subjects with negative nasopharyngeal RT-PCR (20) and serology by RDT (SureScreen). Eligible subjects entered the study *via* three recruitment pathways: (i) active surveillance in 22 Summer schools, as previously reported (21), (ii) passive detection of cases coming from other school-like environments, referred from the Catalonian health surveillance system call, and (iii) individual cases referred from an announcement made to enroll children with positive RT-PCR in the previous 5 days. Symptomatic children were defined as those with acute respiratory infection including fever, cough, headache, gastrointestinal symptoms, rhinorrhea or nasal congestion, anosmia or ageusia, dyspnea, and myalgia. There were 70 adults (25 males and 45 females) and 52 children (26 males and 26 females) recruited. Participants were followed up for 5 weeks over July 2020, with weekly blood and saliva sample collection and RT-PCR testing that allowed accurately defining the infected positive and negative groups. Saliva samples were collected with Oracol devices (Malvern Medical Development, UK) for optimal harvesting of crevicular fluid, enriched with serum antibodies (22, 23). Blood samples were collected by venipuncture and plasma or serum separated by centrifugation and frozen on the same day, or as dried blood spots (DBS) to facilitate the field survey logistics. Blood EDTA tubes were kept in the fridge until centrifugation at 1721g for 10 min to separate the plasma fraction. DBS were stored at -20°C until the day of serum extraction, when they were brought up to room temperature, and mixed with elution buffer (Luminex buffer with 0.05% Tween-20) at 4°C overnight with agitation at 600 rpm. On the next day, tubes were centrifuged at 10,000 rpm for 10 min to harvest the serum. Plasma and DBS serum eluate samples were stored at -80°C until serological analysis. Oracol devices were kept and transported refrigerated to the lab on the same day, for centrifugation into a cryotube at 1500g for 10 min, heat inactivated at 55°C for 30 min, and frozen at -20°C until antibody measurements. Thirty-six pre-pandemic plasmas from healthy adults were used as negative controls and to calculate the seropositivity cutoff for sera/plasmas. No pre-pandemic saliva samples were available after contacting national biobanks.

### Measurement of Antibodies

Quantitative suspension array technology (qSAT) assays to measure IgM, IgA and IgG against SARS-CoV-2 were adapted from our previous standardized serum/plasma protocols (24) to a saliva matrix for SARS-CoV-2 antibody evaluation. Antigens included the nucleocapsid (N) full-length (FL) and C-terminus (amino acid residues 340-416, CT) (25), the spike (S) FL produced at CRG, S2 purchased from SinoBiologicals, and RBD donated by F. Krammer (Mount Sinai, NY). Briefly, proteins coupled to magnetic microspheres (Luminex Corporation, Austin, TX) were incubated with serum/plasma (1/500 dilution) or saliva samples (1/5 or 1/10 dilutions, **Figure S1**) or blank controls in 384-well plates. Saliva dilution of 1/10 showed higher sensitivity and was selected for further assays. The impact of heat inactivation was previously checked in serum/plasma and saliva (**Figure S2**), with a decrease in the levels of IgM to RBD and S in saliva. Serum was eluted from DBS with 200 μl of PBS-BN (filtrated PBS with 1% BSA and 0.05% sodium azide, MilliporeSigma, St. Louis, USA) + 0.05% Tween20. Considering a hematocrit of 50% results in an eluted protein concentration equivalent to a serum/plasma dilution of 1:50, which was subsequently diluted to 1:500 for the assay. Antibodies in serum eluted from DBS and from serum/plasma samples are shown together since no differences were observed in the available paired samples of plasma and DBS. After antigen-coupled beads were incubated with samples, plates were washed and phycoerythrin-labeled secondary antibodies (anti-human IgG, IgM, or IgA, Moss) added. Finally, beads were washed, resupended and acquired in a FlexMap 3D xMAP^®^ instrument. Crude median fluorescent intensities (MFI) and background fluorescence from blank wells were exported using the xPONENT software.

### Data Analysis

To verify the distribution of the data, the Shapiro–Wilk test was applied. Non-parametric Mann-Whitney U tests were used in boxplots to compare levels (log_10_MFI) of each antibody/antigen pair between study groups. Radar charts were used to compare median log_10_MFI of all antibodies together between study groups by Mann-Whitney U test. Heatmaps with hierarchical clustering (Euclidean method) were used to evaluate patterns of responses at the individual level depending on clinical and demographic variables. Due to the unavailability of pre-pandemic saliva samples, we explored calculating seropositivity cutoffs by the mean plus 3 standard deviations (SD) of pandemic negative samples for serum/plasma and for saliva samples (**Figure S3**). All analyses were performed at 5% significance level with R software version 4.0.2. The ggplot2 package was used to perform boxplot graphs (26).

## Results

The basic demographic characteristics of SARS-CoV-2 infected (n=62, including 42 children) and non-infected (n=60) individuals in whom saliva and/or serum/plasma samples were analyzed, are shown in **Table 1**. The full database included in this study is available in **Table S1**.

**Table 1:** Characteristics of study participants from whom samples were analyzed.

	Negatives		Positives	
	Serum^a^	Saliva	Serum^b^	Saliva
	(N = 48)	(N = 61)	(N = 58)	(N = 56)
Age				
Children	5 (10.4%)	10 (16.4%)	40 (69.0%)	40 (71.4%)
Adults	43 (89.6%)	51 (83.6%)	18 (31.0%)	16 (28.6%)
Sex				
Male	15 (31.2%)	19 (31.1%)	30 (51.7%)	28 (50.0%)
Female	33 (68.8%)	42 (68.9%)	28 (48.3%)	28 (50.0%)
Symptoms				
Yes	2 (4.2%)	2 (3.3%)	26 (44.8%)	26 (46.4%)
No	46 (95.8%)	59 (96.7%)	32 (55.2%)	30 (53.6%)
Sample collection (weeks)				
1	4 (20.0%)	9 (34.6%)	17 (32.1%)	19 (34.5%)
2	13 (65.0%)	13 (50.0%)	12 (22.6%)	12 (21.8%)
3	3 (15.0%)	4 (15.4%)	17 (32.1%)	17 (30.9%)
4	0 (0.0%)	0 (0.0%)	6 (11.3%)	6 (10.9%)
5	0 (0.0%)	0 (0.0%)	1 (1.9%)	1 (1.8%)

Adult: age 15 years or older. There were 122 individuals participating in the study, 70 adults (25 males and 45 females) and 52 children (26 males and 26 females). There were 29 symptomatic and 93 asymptomatic individuals. Some individuals only contributed with serum or saliva samples.

a Sixteen serum samples were obtained from dried blood spots (DBS).

b Eight serum samples were obtained from DBS.

### Antibody Levels According to SARS-CoV-2 RT-PCR Status

**Figure 1:**
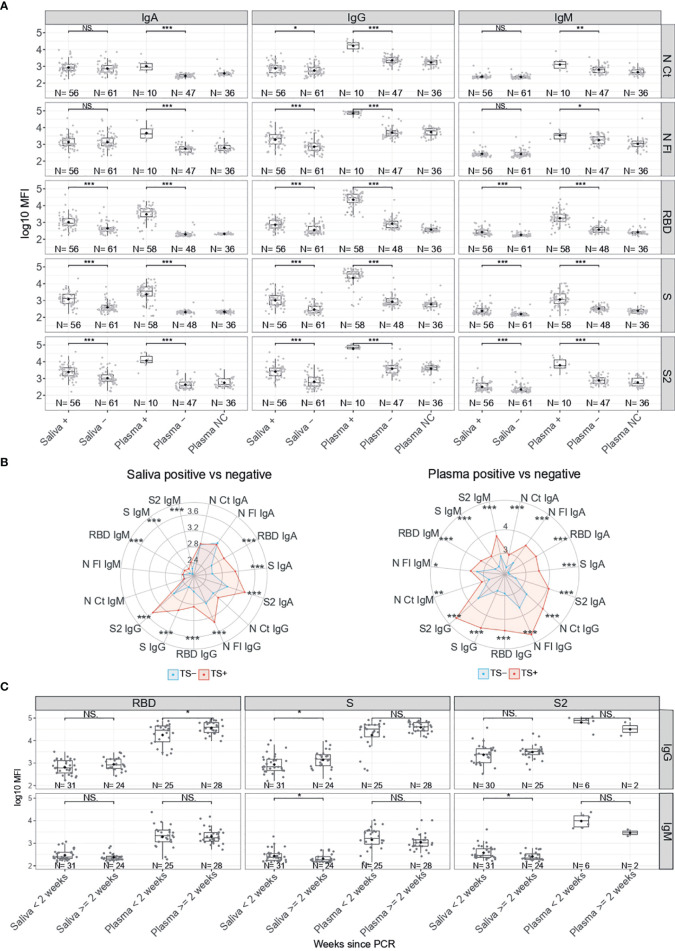
Antibody levels according to SARS-CoV-2 RT-PCR status. **(A)** Boxplots showing log_10_MFI antibody levels. Saliva samples were tested heat inactivated and at 1/10 dilution, and serum (from plasma samples or dry blood spots) at 1/500. **(B)** Radar charts representing the median of the log_10_MFI antibodies in plasma and saliva. TS-: Negative Test Sample, represented in blue. TS+: Positive Test Sample, represented in red. **(C)** Boxplots showing log_10_MFI antibody levels by time since positive RT-PCR. Median log_10_MFI levels were compared by Mann-Whitney U test. Statistically significant raw p-values are highlighted with asterisks. ***p < 0.001, **p < 0.01, *p < 0.05, NS, Not significant.

We compared the levels (log_10_MFI) of IgM, IgA and IgG antibodies to five SARS-CoV-2 antigens in saliva and serum/plasma samples from RT-PCR positive and negative individuals. IgG levels to all antigens in saliva were statistically significantly higher (p<0.05 for N CT and p<0.001 for the rest) in RT-PCR positive than negative individuals (**Figures 1A, B**). Levels of IgM and IgA to S (FL, S2 and RBD) but not N (FL, CT) antigens in saliva were statistically significantly higher in RT-PCR positive than negative individuals (p<0.001). In serum/plasma samples from the same individuals, all Ig isotypes were significantly higher in positive than negative individuals (p<0.05 for IgM to N and p<0.001 for the rest). The magnitude of antibody responses was substantially lower in saliva than serum/plasma samples despite being measured at a higher concentration (1/10 vs 1/500), and levels overlapped between positive and negative individuals to a higher degree in saliva than serum/plasma samples (**Figure S3**).

We evaluated whether RT-PCR positive individuals who were antibody negative had more recent infections. Stratified by time since diagnosis, levels of IgG against S in saliva and against RBD in serum/plasma were higher in samples collected >2 weeks after positive RT-PCR (p<0.05) (**Figure 1C**). In contrast, levels of IgM to S and S2 in saliva were lower in samples collected >2 weeks after positive RT-PCR (p<0.05).

### Antibody Levels by Age and Sex According to SARS-CoV-2 RT-PCR Status

**Figure 2:**
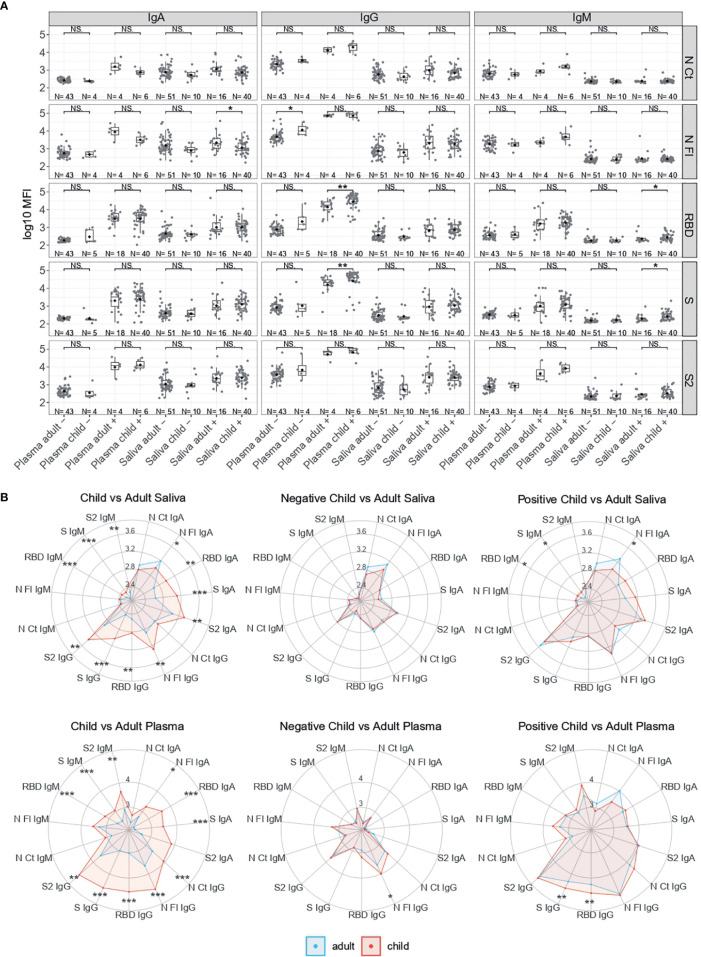
Antibody levels according to SARS-CoV-2 RT-PCR status and by age. **(A)** Boxplots showing log_10_MFI antibody levels of saliva samples tested heat inactivated and at 1/10 dilution, and serum (from plasma samples or dry blood spots) at 1/500. **(B)** Radar charts comparing the medians of antibody levels (in log_10_MFI) between child and adult plasma and saliva samples Adults are represented in blue and children in red. Median log_10_MFI antibody levels were compared by Mann-Whitney U test. Statistically significant raw p-values are highlighted with asterisks. ***p < 0.001, **p < 0.01, *p < 0.05, NS, not significant.

Among infected individuals, children had significantly higher serum/plasma levels of IgG to RBD and S (p<0.01), and significantly higher saliva levels of IgM to RBD and S than adults (p<0.05) (**Figure 2**). In contrast, infected adults had significantly higher levels of IgA to N FL in saliva than infected children (p<0.05). Infected male children also had higher saliva levels of IgG to N CT, N FL and RBD than female children (p<0.05) (**Figure S4**). Among SARS-CoV-2 RT-PCR negative individuals, children compared to adults had significantly higher serum/plasma levels of IgG to N FL (p<0.05).

### Antibody Levels According to Presence/Absence of Symptoms

**Figure 3:**
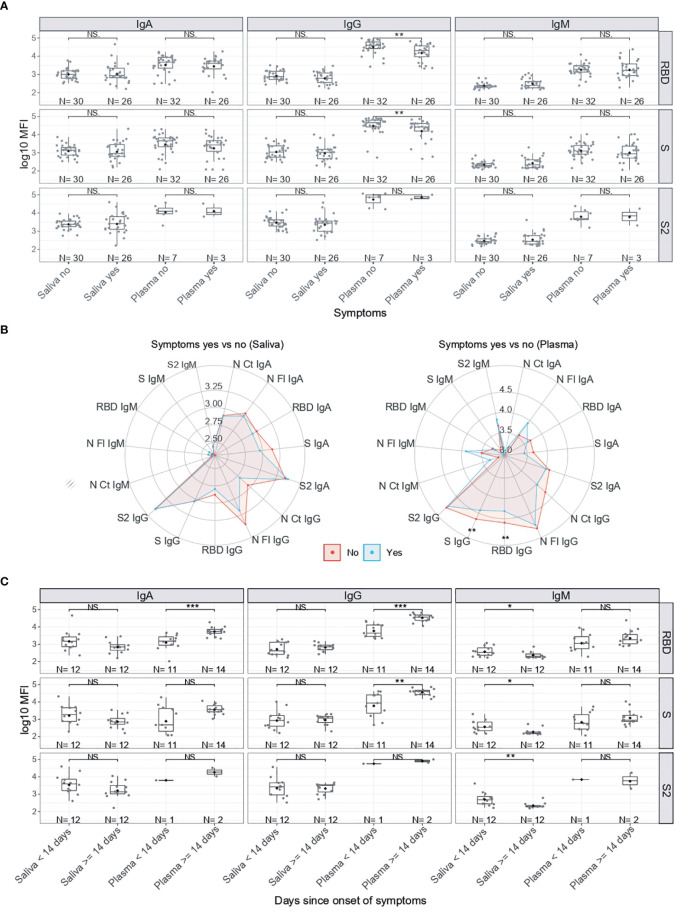
Antibody levels by symptoms in SARS-CoV-2 positive individuals. **(A)** Boxplots showing log_10_MFI antibody levels in saliva samples tested heat inactivated and at 1/10 dilution, and serum (from plasma samples or dry blood spots) at 1/500. **(B)** Radar charts comparing the medians of antibody levels (in log_10_MFI) in serum/plasma and saliva between symptomatic (blue) and asymptomatic (red) individuals. **(C)** Boxplots showing log_10_MFI antibody levels in saliva and plasma samples by Time since onset of symptoms. Median log_10_MFI antibody levels were compared by Mann-Whitney U test. Statistically significant raw p-values are indicated with asterisks. ***p < 0.001, **p < 0.01, *p < 0.05, NS: not significant.

Among RT-PCR positive individuals, IgG levels to RBD and S were significantly higher in serum/plasma from asymptomatic than symptomatic subjects (p<0.01) (**Figure 3A**). In general, IgG and IgA but not IgM tended to be lower in saliva and serum/plasma from SARS-CoV-2 infected subjects who developed symptoms (**Figure 3B**). Stratifying by time since symptoms onset, serum IgA and IgG to RBD and IgG to S were higher 14 days after the onset of symptoms (p<0.01). In contrast, IgM to RBD, S and S2 in saliva were lower 14 days after the onset of symptoms (p<0.05) (**Figure 3C**).

### Correlation of Antibody Levels Between Saliva and Serum/Plasma Samples

**Figure 4:**
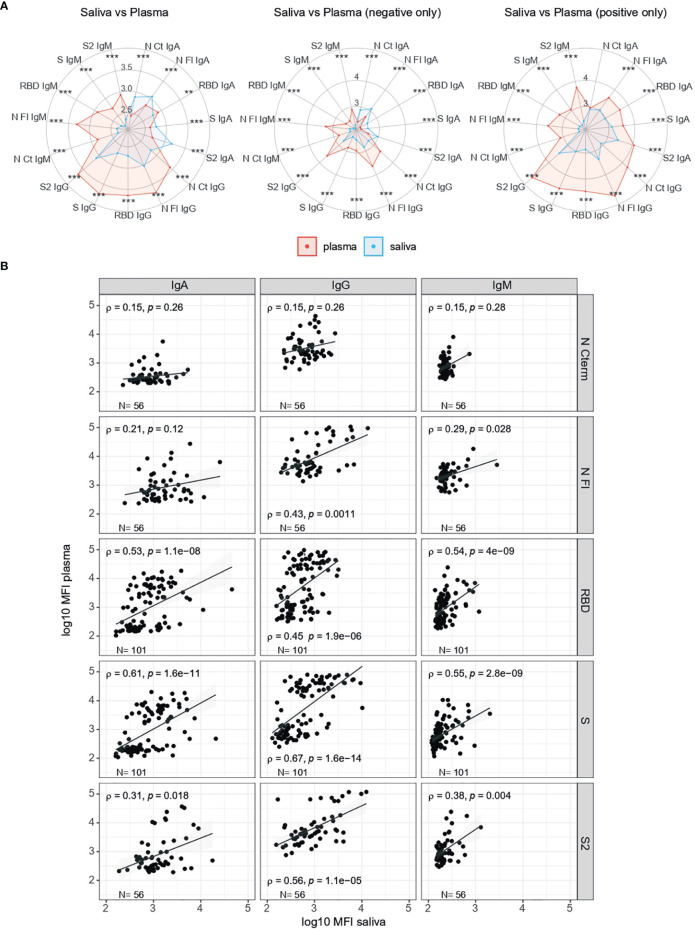
Comparison of antibody levels in serum/plasma and saliva samples. **(A)** Radar charts comparing the medians of antibody levels (in log_10_MFI) in serum/plasma (red) and saliva (blue), overall and by RT-PCR status. **(B)** Correlations of isotype-antigen pair levels between plasma and saliba samples. X axis shows the saliva levels at 1/10 dilution, inactivated; Y axis show serum/plasma levels at 1/500 dilution, not inactivated. Median log_10_MFI antibody levels were compared by Mann-Whitney U test. Statistically significant raw p-values are highlighted with asteriks. ***p < 0.001, **p < 0.01.

The pattern of antibody responses in serum/plasma versus saliva samples varied depending on the Ig isotype and antigen (**Figure 4A**). Relative antibody levels in RT-PCR positive individuals were higher in serum/plasma than saliva samples, but in RT-PCR negatives, IgA levels were higher in saliva than serum/plasma samples. There was a statistically significant correlation between serum/plasma and saliva levels for all antibody isotypes against S antigens (p<0.01) and also for IgG and IgM to N FL (p<0.05) (**Figure 4B**). The strongest correlations were for IgG and IgA to S, followed by IgG to S2, and IgA and IgM to RBD.

### Multimarker Analysis of Antibody Responses

**Figure 5:**
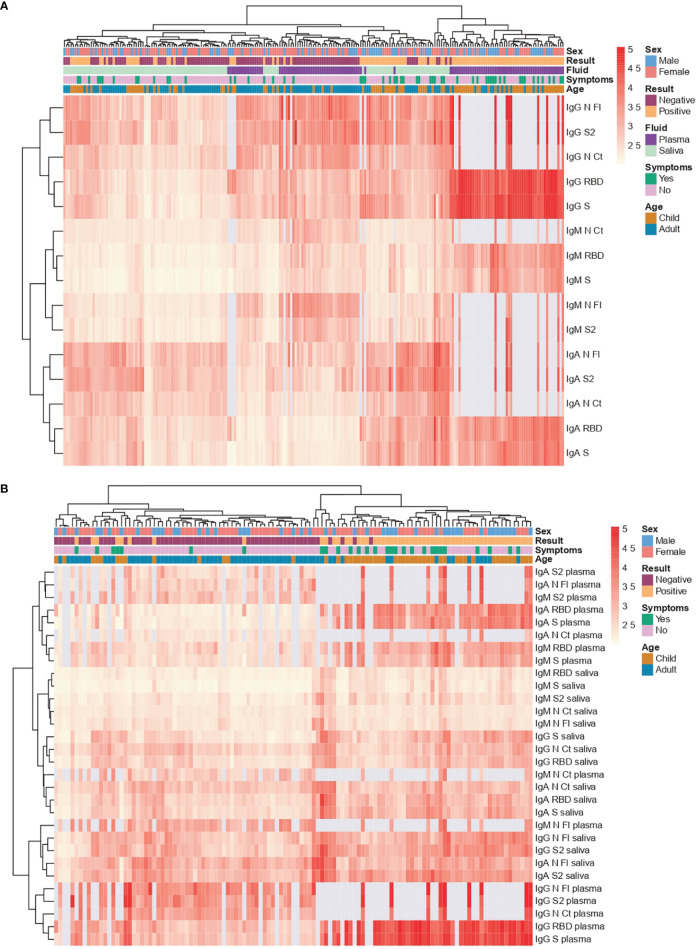
Heatmap with hierarchical clustering. Saliva samples were tested heat inactivated and at 1/10 dilution, and serum (from plasma samples or dry blood spots) at 1/500. **(A)** Per sample (each column) **(B)** Per individual (each column). Light grey represents missing data, as more samples were tested for S and RBD than for the rest of antigens.

Combining all the antibody isotype and antigen responses per sample (**Figure 5A**) and per individual (**Figure 5B**), and considering RT-PCR status, age and symptoms, a hierarchical clustering heatmap analysis revealed different patterns. Most samples from RT-PCR positive individuals clustered together (**Figures 5A, B**) and clusters by type of samples (saliva or serum/plasma) were also observed (**Figure 5A**). RT-PCR positive individuals tended to have a wider breadth of high-level antibody responses (right side) particularly intense anti-S and anti-RBD responses in serum/plasma samples, but clusters of high responses also mapped with RT-PCR negative individuals, including some N FL, N CT and S2 IgG and IgM serum/plasma (center **Figure 5A**) or IgA saliva (left **Figure 5A**) responders. Intensity of responses was generally lower for IgM particularly in saliva, which could be influenced by the inactivation. No clear clustering was observed according to symptoms, while higher responses appeared to predominate more in children than adults.

## Discussion

We showed that significantly higher levels of saliva antibodies to SARS-CoV-2 could be measured from RT-PCR-confirmed cases than from RT-PCR-negative individuals with our high-throughput multiplex qSAT assays. Most antibody responses correlated significantly between saliva and serum/plasma samples but Spearman coefficients were moderate/moderate-high (rho<0.7) and depended on the Ig isotype and antigen pair. These variable correlations would indicate different dynamics of antibody responses in blood versus mucosal tissues (18, 27, 28). This suggests that responses that may not be detected in serum/plasma could be patent in saliva, and vice versa (29). This makes the saliva antibody assay relevant and complementary to serology assays. The fact that saliva antibody levels were much lower than blood levels but considering that saliva is a non-invasive and easy to use approach in the field compared to nasal swabs or blood pricking, represents a trade off between accuracy versus quicker and wider deployability that makes it valuable for pediatric studies.

We examined potential reasons for less discrimination between infected and non-infected subjects in saliva than in serum/plasma samples. On one hand, low antibody levels in some infected individuals could be due to non-responsiveness (30) or because a too recent exposure, as seen by higher IgGs two weeks after RT-PCR diagnosis. Low levels in saliva may not be explained by inappropriate sample collection as we used the Oracol devices that yield higher titres of total antibodies compared with other saliva/oral fluid sampling methods (31) and are well accepted across age groups (32). However, the quantity of antibody levels measured could have been affected by heat inactivation (18), therefore other methods based on Triton X-100 incubation would be preferred. On the other hand, medium-high Ig levels in saliva samples from RT-PCR negative and/or serum/plasma seronegative individuals could be indicative of a previous or current exposure to SARS-CoV-2 that was not detected by RT-PCR or serology (done by less sensitive RDT methods), i.e. false negatives. Indeed, being a study that recruited contacts of RT-PCR positive cases, it is not unreasonable that saliva serology could be more sensitive to detect infections with low or fast-resolving viral loads that might only induce a local mucosal response able to control viremia without the need to elicit a systemic response (33). In those cases, SARS-CoV-2-specific serum IgA titers may last shorter whereas serum IgG titers might remain negative or become positive later after symptom onset while mucosal IgA might be more patent. Thus, in addition to serum IgA and IgG, measurement of SARS-CoV-2-specific saliva IgA should be considered to better estimate the percentage of individuals who have experienced coronavirus infection (34). Alternatively, the detection of SARS-CoV-2 antibodies in saliva of RT-PCR negative people could relate to cross-reactivity to common cold HCoV that are more common in children than in adults. Higher levels of pre-existing IgG to HCoV have been proposed as one potential explanation for the lower COVID-19 incidence in children (7, 8). We have seen that antibodies to N FL, followed by S2, are more present in pre-pandemic samples and more cross-reactive (25). These antibodies may also be more prevalent in saliva than in serum/plasma.

We investigated other factors that could be associated with the antibody responses in saliva. We observed different patterns to what is reported in cross-sectional population studies, where adults and symptomatic individuals tend to have higher antibody levels than children and asymptomatic ones, respectively. This could be related to the population under study, who are mostly children infected cases and their contacts. Higher levels of anti-S IgG among asymptomatic individuals could indicate protection against disease in infected individuals, and higher IgG and IgM levels in children could also be related to a stronger immunity. High antibody titers in saliva in vaccinated individuals have been related to lower transmission potential (35). The levels of antibodies in relation to days after positive RT-PCR or days since symptoms onset reflect the kinetics whereby saliva IgM are the first to appear and decay, while serum IgA and IgG increase later. Lower viral load has been associated with faster antibody kinetics (36). In relation to sex, the higher levels of saliva IgG to N and RBD in RT-PCR positive male children than female children could reflect sex-related differences in viral load (marker of exposure) or the ability to induce better immunity (marker of protection). This pattern is in contrast with serological studies in adults that did not find differences in males and females with mild or no symptoms (37), and could again reflect disparate dynamics of mucosal versus systemic responses, potentially affected by sex.

The significant role of mucosal immunity and, particularly, of secretory and circulating IgA antibodies in COVID-19, is becoming more apparent, and could be exploited for beneficial diagnostic, therapeutic, or prophylactic purposes (vaccines) (14). This supports the importance for screening antibodies in saliva in addition to serum. There is increasing evidence in favor of a key role for IgA in early virus neutralization (38): (i) early SARS-CoV-2-specific responses are typically dominated by the IgA isotype, (ii) peripheral expansion of IgA-plasmablasts with mucosal-homing shortly after the onset of symptoms and peak during the third week of the disease, and (iii) IgA may contribute to a much larger extent to virus neutralization as compared to IgG (28, 39).

A study limitation was that seropositivity thresholds could not be estimated with pre-pandemic saliva samples due to lack of access to them, and thus sensitivity and specificity could not be established for the saliva assays by standard methods. Using RT-PCR negative pandemic samples was somewhat useful for serum/plasma samples, but with saliva there was substantial overlap between antibody levels in infected and non-infected individuals. In addition, being pandemic samples, we cannot ascertain that they were not previously exposed at low levels and therefore this approach is not optimal. This constraint could be overcome in follow up studies by assessing seroconversion in consecutive samples calculating the fold change increase in levels (e.g. ≥4) over a given study period (21, 28) and, in future, access to pre-pandemic samples in international biobanks will be sought. Finally, the sample size was limited to establish robust associations between factors affecting antibody levels, and there could be some imbalance between age and infection that might affect the results, therefore data should be interpreted with caution and need to be confirmed in larger studies. However, we analyzed the effect of age stratifying by infection status to take potential imbalances into account.

In conclusion, antibody levels in saliva measured with our high-throughput qSAT assays largely correlated with those in serum/plasma from individuals with confirmed RT-PCR diagnosis of SARS-CoV-2 infection, but the degree of correlation depended on the isotype and antigen. Higher antibody levels found in asymptomatic individuals and in children, particularly among males, could indicate protection against disease and stronger immunity. This non-invasive field deployable antibody screening could be useful to establish the percentage of people who have been exposed to SARS-CoV-2 in epidemiological surveys, and to assess the longitudinal maintenance of antibodies (40), particularly in children who were not initially prioritized in 2021 for COVID-19 vaccination. Furthermore, considering the significant correlation between saliva and serum/plasma of antibody levels to S, it could also be an attractive tool to monitor induction and maintenance of vaccine responses, including to variants of concern, as part of large-scale immunization campaigns (35, 41–43).

## Data Availability Statement

The original contributions presented in the study are included in the article/**Supplementary Material**. Further inquiries can be directed to the corresponding authors.

## Ethics Statement

The studies involving human participants were reviewed and approved by Institutional Review Board and the Hospital Sant Joan de Déu Ethics Committee (Ref. PIC 153-20) and the Hospital Clínic ethics committee (Ref. CEIC-7455). Written informed consent to participate in this study was provided by the participants’ legal guardian/next of kin.

## Author Contributions

CD and SAl wrote the first draft of the manuscript. RA, GM, and IJ contributed to the manuscript write up. NR, CC, and LI produced proteins for immunoassays. DB, LP, and AC managed samples. MV, AJ, RR, and SAl performed antibody assays. RA, GM, and CD coordinated immunology and data analysis. RS and SAl performed data analysis. CMA performed microbiology analyses. GP, MM, MH, MG-A, AG-M, EB-C, JC, MC, CF, VF, QB, BB, PM-M, SAj, NB, SAr, MF, EG, JG-G, and IJ performed the clinical and epidemiological studies. All read approved the final version of the manuscript.

## Funding

The project has been funded by Stavros Niarchos Foundation (SNF), Banco Santander and other private donors of KidsCorona, and Fundació Privada Daniel Bravo Andreu. RR had the support of the Health Department, Catalan Government (PERIS SLT017/20/000224). Development of SARS-CoV-2 reagents was partially supported by the NIAID Centers of Excellence for Influenza Research and Surveillance (CEIRS) contract HHSN272201400008C. ISGlobal receives support from the Spanish Ministry of Science and Innovation through the “Centro de Excelencia Severo Ochoa 2019-2023” Program (CEX2018-000806-S), and support from the Generalitat de Catalunya through the CERCA Program. CISM is supported by the Government of Mozambique and the Spanish Agency for International Development (AECID).

## Conflict of Interest

The authors declare that the research was conducted in the absence of any commercial or financial relationships that could be construed as a potential conflict of interest.

## Publisher’s Note

All claims expressed in this article are solely those of the authors and do not necessarily represent those of their affiliated organizations, or those of the publisher, the editors and the reviewers. Any product that may be evaluated in this article, or claim that may be made by its manufacturer, is not guaranteed or endorsed by the publisher.

## References

[1] Mehta NS Mytton OT Mullins EWS Fowler TA Falconer CL Murphy OB. SARS-Cov-2 (COVID-19): What Do We Know About Children? A Systematic Review. Clin Infect Dis (2020) 71(9):2469–79. doi: 10.1093/cid/ciaa556 PMC7239259 32392337

[2] Smith BK Janowski AB Danis JE Harvey IB Zhao H Dai Y-N. Seroprevalence of SARS-Cov-2 Antibodies in Children and Adults in St. Louis, Missouri, USA. mSphere (2021) 6(1):e01027–20. doi: 10.1128/mSphere.01207-20 PMC7860990 33536325

[3] Rytter MJH Nygaard U Mandic IN Glenthøj JP Schmidt LS Cortes D. Prevalence of SARS-Cov-2-Antibodies in Danish Children and Adults. Pediatr Infect Dis J (2021) 40(4):e157–9. doi: 10.1097/INF.0000000000003048 33427800

[4] Waterfield T Watson C Moore R Ferris K Tonry C Watt A. Seroprevalence of SARS-Cov-2 Antibodies in Children: A Prospective Multicentre Cohort Study. Arch Dis Child (2021) 106(7):680–6. doi: 10.1136/archdischild-2020-320558 33172887

[5] Zinszer K McKinnon B Bourque N Pierce L Saucier A Otis A. Seroprevalence of SARS-Cov-2 Antibodies Among Children in School and Day Care in Montreal, Canada. JAMA (2021) 4(11):e2135975. doi: 10.1001/jamanetworkopen.2021.35975 PMC8611475 34812845

[6] Zimmermann P Curtis N. COVID-19 in Children, Pregnancy and Neonates: A Review of Epidemiologic and Clinical Features. Pediatr Infect Dis J (2020) 39(6):469–77. doi: 10.1097/INF.0000000000002700 PMC7363381 32398569

[7] Khan T Rahman M Al AF SSY H Ata M Zhang Q. Distinct Antibody Repertoires Against Endemic Human Coronaviruses in Children and Adults. JCI Insight (2021) 6(4):e144499. doi: 10.1172/jci.insight.144499 PMC7934927 33497357

[8] Nogrady B. How Kids’ Immune Systems can Evade COVID [Internet]. Nat.England (2020) 588:382. doi: 10.1038/d41586-020-03496-7 33303982

[9] Pavel AB Wu J Renert-Yuval Y Del Duca E Glickman JW Miller RL. SARS-Cov-2 Receptor ACE2 Protein Expression in Serum Is Significantly Associated With Age. Allergy (2020) 76(3):875–8. doi: 10.1111/all.14522. Denmark. PMC8278339 32726474

[10] Posfay-Barbe KM Wagner N Gauthey M Moussaoui D Loevy N Diana A. COVID-19 in Children and the Dynamics of Infection in Families. Pediatrics (2020) 146(2):e20201576. doi: 10.1542/peds.2020-1576 32457213

[11] Long Q-X Tang X-J Shi Q-L Li Q Deng H-J Yuan J. Clinical and Immunological Assessment of Asymptomatic SARS-Cov-2 Infections. Nat Med (2020) 26(8):1200–4. doi: 10.1038/s41591-020-0965-6 32555424

[12] Grossberg AN Koza LA Ledreux A Prusmack C Krishnamurthy HK Jayaraman V. A Multiplex Chemiluminescent Immunoassay for Serological Profiling of COVID-19-Positive Symptomatic and Asymptomatic Patients. Nat Commun (2021) 12(1):740. doi: 10.1038/s41467-021-21040-7 33531472 PMC7854643

[13] Lisboa Bastos M Tavaziva G Abidi SK Campbell JR Haraoui L-P Johnston JC. Diagnostic Accuracy of Serological Tests for Covid-19: Systematic Review and Meta-Analysis. BMJ (2020) 370:m2516. doi: 10.1136/bmj.m2516 32611558 PMC7327913

[14] Russell MW Moldoveanu Z Ogra PL Mestecky J. Mucosal Immunity in COVID-19: A Neglected But Critical Aspect of SARS-Cov-2 Infection. Front Immunol (2020) 11:3221. doi: 10.3389/fimmu.2020.611337 PMC7733922 33329607

[15] Pisanic N Randad PR Kruczynski K Manabe YC Thomas DL Pekosz A. COVID-19 Serology at Population Scale: SARS-Cov-2-Specific Antibody Responses in Saliva. J Clin Microbiol (2020) 59(1):e02204–20. doi: 10.1128/JCM.02204-20 PMC7771435 33067270

[16] Heinzel C Pinilla YT Elsner K Friessinger E Mordmüller B Kremsner PG. Non-Invasive Antibody Assessment in Saliva to Determine SARS-Cov-2 Exposure in Young Children. Front Immunol (2021) 12:4203. doi: 10.3389/fimmu.2021.753435 PMC8531807 34691072

[17] To KK-W Tsang OT-Y Leung W-S Tam AR Wu T-C Lung DC. Temporal Profiles of Viral Load in Posterior Oropharyngeal Saliva Samples and Serum Antibody Responses During Infection by SARS-Cov-2: An Observational Cohort Study. Lancet Infect Dis (2020) 20(5):565–74. doi: 10.1016/S1473-3099(20)30196-1 PMC7158907 32213337

[18] Isho B Abe KT Zuo M Jamal AJ Rathod B Wang JH. Persistence of Serum and Saliva Antibody Responses to SARS-Cov-2 Spike Antigens in COVID-19 Patients. Sci Immunol (2020) 5(52):eabe5511. doi: 10.1126/sciimmunol.abe5511 33033173 PMC8050884

[19] Heaney CD Pisanic N Randad PR Kruczynski K Howard T Zhu X. Comparative Performance of Multiplex Salivary and Commercially Available Serologic Assays to Detect SARS-Cov-2 Igg and Neutralization Titers. J Clin Virol (2021) 145:104997. doi: 10.1016/j.jcv.2021.104997 34695724 PMC8502080

[20] Jordan I de Sevilla MF Fumado V Bassat Q Bonet-Carne E Fortuny C. Transmission of SARS-Cov-2 Infection Among Children in Summer Schools Applying Stringent Control Measures in Barcelona, Spain. Clin Infect Dis (2021) 74(1):66–73. doi: 10.1093/cid/ciab227 PMC7989514 33709138

[21] Dobaño C Alonso S Fernández de Sevilla M Vidal M Jiménez A Pons Tomas G. Antibody Conversion Rates to SARS-Cov-2 in Saliva From Children Attending Summer Schools in Barcelona, Spain. BMC Med (2021) 19(1):309. doi: 10.1186/s12916-021-02184-1 34809617 PMC8608564

[22] Nishanian P Aziz N Chung J Detels R Fahey JL. Oral Fluids as an Alternative to Serum for Measurement of Markers of Immune Activation. Clin Diagn Lab Immunol (1998) 5(4):507–12. doi: 10.1128/CDLI.5.4.507-512.1998 PMC95609 9665958

[23] McKie A Vyse A Maple C. Novel Methods for the Detection of Microbial Antibodies in Oral Fluid. Lancet Infect Dis (2002) 2(1):18–24. doi: 10.1016/S1473-3099(01)00169-4 11892490

[24] Dobaño C Vidal M Santano R Jiménez A Chi J Barrios D. Highly Sensitive and Specific Multiplex Antibody Assays to Quantify Immunoglobulins M, a and G Against SARS-Cov-2 Antigens. J Clin Microbiol (2020) 59(2):e01731–20. doi: 10.1128/JCM.01731-20 PMC8111153 33127841

[25] Dobaño C Santano R Jiménez A Vidal M Chi J Rodrigo Melero N. Immunogenicity and Crossreactivity of Antibodies to the Nucleocapsid Protein of SARS-Cov-2: Utility and Limitations in Seroprevalence and Immunity Studies. Transl Res (2021), S1931–5244(21)00029-3. doi: 10.1016/j.trsl.2021.02.006 PMC7879156 33582244

[26] Wickham H. Ggplot2: Elegant Graphics for Data Analysis [Internet]. New York: Springer-Verlag New York (2016). Available at: https://ggplot2.tidyverse.org.

[27] Griffin SM Chen IM Fout GS Wade TJ Egorov AI. Development of a Multiplex Microsphere Immunoassay for the Quantitation of Salivary Antibody Responses to Selected Waterborne Pathogens. J Immunol Methods (2011) 364(1–2):83–93. doi: 10.1016/j.jim.2010.11.005 21093445

[28] Wade TJ Griffin SM Egorov AI Sams E Hudgens E Augustine S. Application of a Multiplex Salivary Immunoassay to Detect Sporadic Incident Norovirus Infections. Sci Rep (2019) 9(1):19576. doi: 10.1038/s41598-019-56040-7 31862970 PMC6925267

[29] Faustini SE Jossi SE Perez-Toledo M Shields A Allen JD Watanabe Y. Detection of Antibodies to the SARS-Cov-2 Spike Glycoprotein in Both Serum and Saliva Enhances Detection of Infection. MedRxiv (2020). doi: 10.1101/2020.06.16.20133025

[30] Oved K Olmer L Shemer-Avni Y Wolf T Supino-Rosin L Prajgrod G. Multi-Center Nationwide Comparison of Seven Serology Assays Reveals a SARS-Cov-2 Non-Responding Seronegative Subpopulation. EClinicalMedicine-The Lancet (2020), 29:100651. doi: 10.1016/j.eclinm.2020.100651 PMC7676374 33235985

[31] Vyse AJ Cohen BJ Ramsay ME. A Comparison of Oral Fluid Collection Devices for Use in the Surveillance of Virus Diseases in Children. Public Health (2001) 115(3):201–7. doi: 10.1016/S0033-3506(01)00444-9 11429716

[32] Nokes DJ Enquselassie F Vyse A Nigatu W Cutts FT Brown DW. An Evaluation of Oral-Fluid Collection Devices for the Determination of Rubella Antibody Status in a Rural Ethiopian Community. Trans R Soc Trop Med Hyg (1998) 92(6):679–85. doi: 10.1016/S0035-9203(98)90811-2 10326122

[33] Tosif S Neeland MR Sutton P Licciardi PV Sarkar S Selva KJ. Immune Responses to SARS-Cov-2 in Three Children of Parents With Symptomatic COVID-19. Nat Commun (2020) 11(1):5703. doi: 10.1038/s41467-020-19545-8 33177504 PMC7658256

[34] Cervia C Nilsson J Zurbuchen Y Valaperti A Schreiner J Wolfensberger A. Systemic and Mucosal Antibody Responses Specific to SARS-Cov-2 During Mild Versus Severe COVID-19. J Allergy Clin Immunol (2021) 147(2):545–57. doi: 10.1016/j.jaci.2020.10.040 PMC7677074 33221383

[35] Becker M Dulovic A Junker D Ruetalo N Kaiser PD Pinilla YT. Immune Response to SARS-Cov-2 Variants of Concern in Vaccinated Individuals. Nat Commun (2021) 12(1):3109. doi: 10.1038/s41467-021-23473-6 34035301 PMC8149389

[36] Silva J Lucas C Sundaram M Israelow B Wong P Klein J. Saliva Viral Load Is a Dynamic Unifying Correlate of COVID-19 Severity and Mortality. MedRxiv (2021). doi: 10.1101/2021.01.04.21249236

[37] Zeng F Dai C Cai P Wang J Xu L Li J. A Comparison Study of SARS-Cov-2 Igg Antibody Between Male and Female COVID-19 Patients: A Possible Reason Underlying Different Outcome Between Gender. MedRxiv (2020). doi: 10.1101/2020.03.26.20040709 PMC7267228 32383183

[38] Sterlin D Mathian A Miyara M Mohr A Anna F Claër L. Iga Dominates the Early Neutralizing Antibody Response to SARS-Cov-2. Sci Transl Med (2021) 13(577):eabd2223. doi: 10.1126/scitranslmed.abd2223 33288662 PMC7857408

[39] Zeng W Ma H Ding C Yang Y Sun Y Huang X. Characterization of SARS-Cov-2-Specific Antibodies in COVID-19 Patients Reveals Highly Potent Neutralizing Iga. Nature (2021) 6(1):35. doi: 10.1038/s41392-021-00478-7 PMC7844101 33514692

[40] Alkharaan H Bayati S Hellström C Aleman S Olsson A Lindahl K. Persisting Salivary Igg Against SARS-Cov-2 at 9 Months After Mild COVID-19: A Complementary Approach to Population Surveys. J Infect Dis (2021) 224(3):407–14. doi: 10.1093/infdis/jiab256 PMC8244549 33978762

[41] Pinilla YT Heinzel C Caminada L-F Consolaro D Esen M Kremsner PG. SARS-Cov-2 Antibodies Are Persisting in Saliva for More Than 15 Months After Infection and Become Strongly Boosted After Vaccination. Front Immunol (2021) 12:5146. doi: 10.3389/fimmu.2021.798859 PMC8695841 34956236

[42] Ketas TJ Chaturbhuj D Cruz-Portillo VM Francomano E Golden E Chandrasekhar S. Antibody Responses to SARS-Cov-2 Mrna Vaccines Are Detectable in Saliva. BioRxiv (2021). doi: 10.1101/2021.03.11.434841 PMC8201795 34136730

[43] Sano K Bhavsar D Singh G Floda D Srivastava K Gleason C. Efficient Mucosal Antibody Response to SARS-Cov-2 Vaccination Is Induced in Previously Infected Individuals. MedRxiv (2021). doi: 10.1101/2021.12.06.21267352

