# Supplementary material for: Multiplex Antibody Analysis of IgM, IgA and IgG to SARS-CoV-2 in Saliva and Serum From Infected Children and Their Close Contacts
Source: Front Immunol. 2022 Jan 27;13:751705. doi: 10.3389/fimmu.2022.751705 (PMC8828491; doi:10.3389/fimmu.2022.751705)
Supplement: Supplementary file 1 [file DataSheet_1.docx]

Supplementary Material

Multiplex Antibody Analysis of IgM, IgA and IgG to SARS-CoV-2 in Saliva and Serum from Infected Children and their Close Contacts

**Carlota Dobaño, Selena Alonso, et al.**

# Supplementary Figures

**Supplementary Figure 1.** Selection of dilution of saliva 1/5 vs. 1/10 in positive and negative test samples (TS).


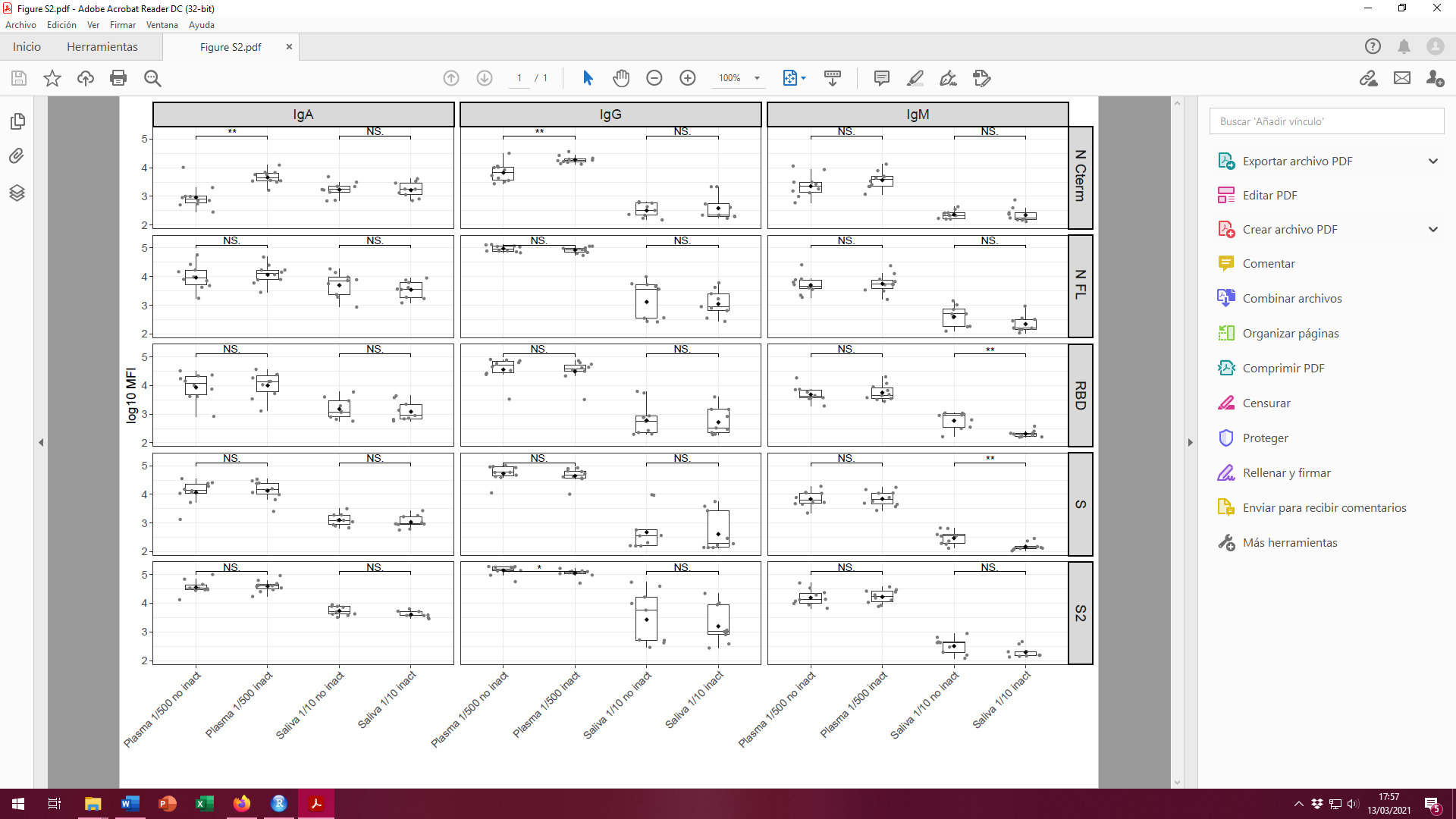


**Supplementary Figure 2.** Effect of heat inactivation in saliva and serum/plasma samples.


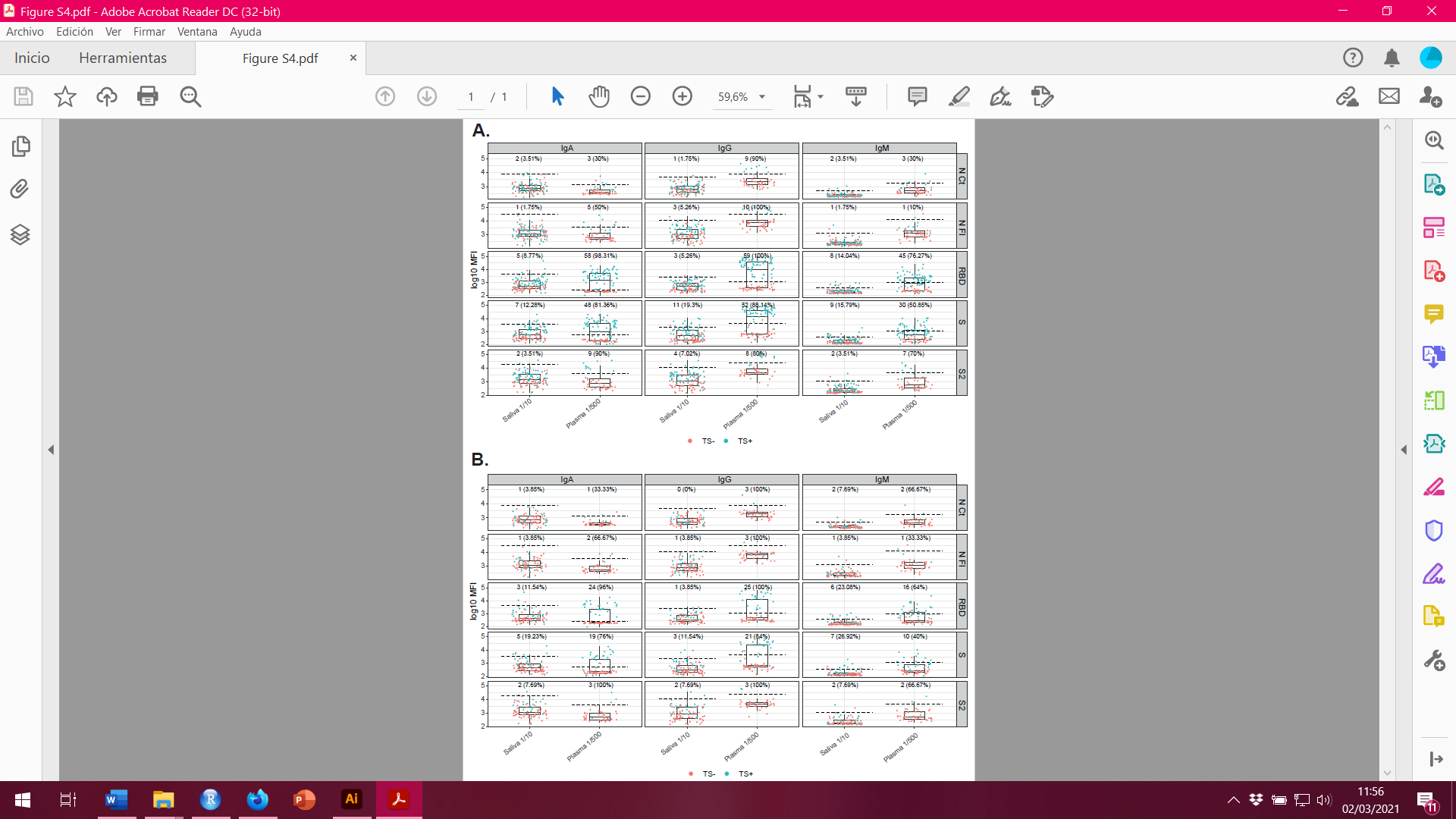


**Supplementary Figure 3. Exploring seropositivity cutoffs calculated with negative pandemic samples.** Cutoffs estimated with SARS-CoV-2 RT-PCR negative pandemic samples (TS-) discriminated up to 100% the positive individuals (TS+) depending on the antigen in serum/plasma but not saliva samples due to the overlap in antibody levels **(A)**. Cutoffs estimated with the RT-PCR negative pandemic samples in symptomatic patients discriminated positive individuals in more antigens **(B)**. Salivas were tested at 1/10 dilution and serum/plasmas at 1/500 dilution.


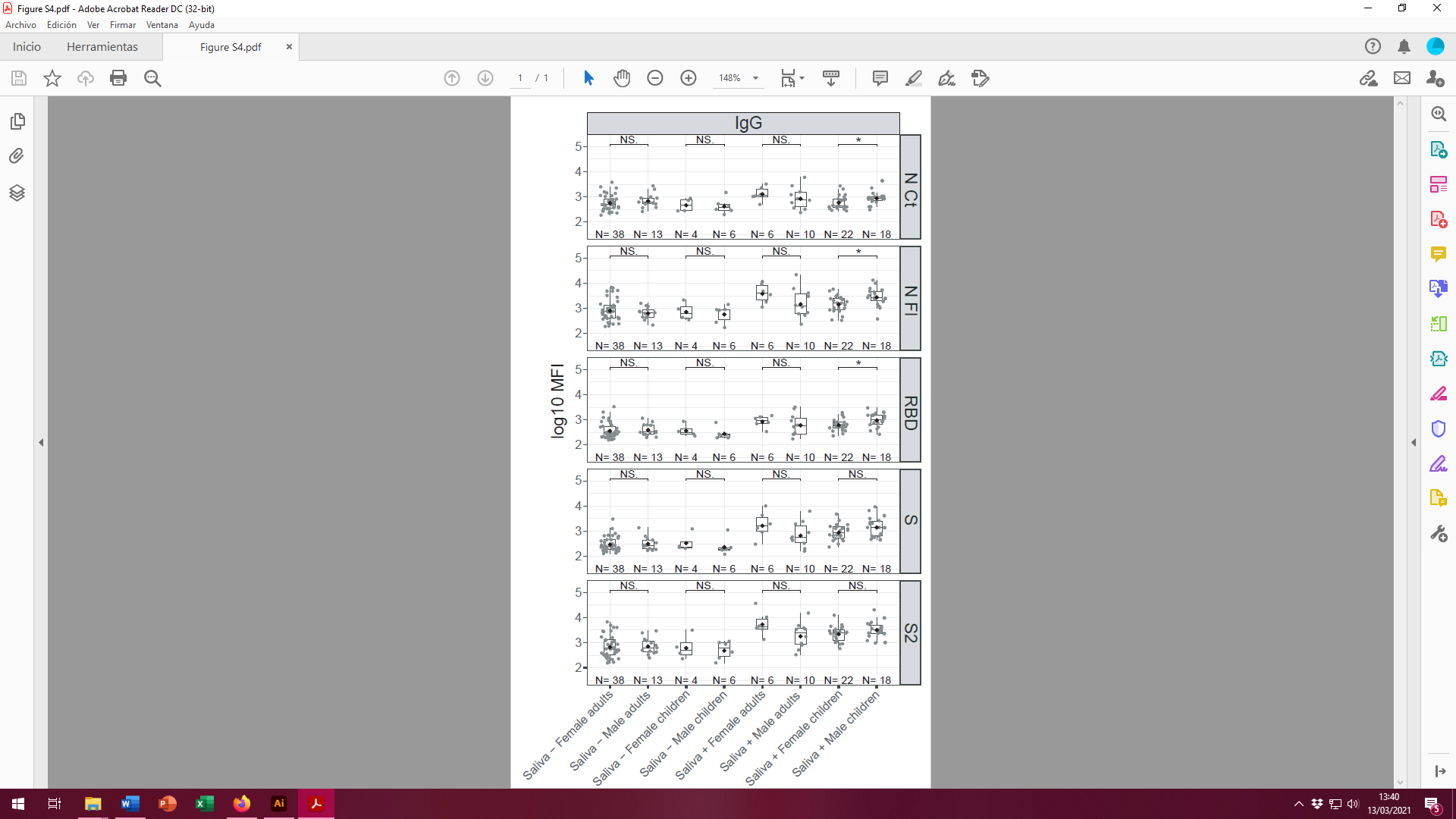


**Supplementary Figure 4.** Comparison of antibody levels in saliva samples stratified by sex and age.
